# A case report of spontaneous abortion caused by *Brucella melitensis* biovar 3

**DOI:** 10.1186/s40249-018-0411-x

**Published:** 2018-05-02

**Authors:** Hong-Xia Yang, Jun-Jun Feng, Qiu-Xiang Zhang, Rui-E Hao, Su-Xia Yao, Rong Zhao, Dong-Ri Piao, Bu-Yun Cui, Hai Jiang

**Affiliations:** 1 Disease Inspection Laboratory, Shanxi Center for Disease Control and Prevention, Taiyuan, China; 2 Clinical Laboratory, Shanxi Dayi Hospital, Taiyuan, China; 3 State Key Laboratory for Infectious Disease Prevention and Control, Collaborative Innovation Center for Diagnosis and Treatment of Infectious Diseases, National Institute for Communicable Disease Control and Prevention, Chinese Center for Disease Control and Prevention, Beijing, China

**Keywords:** *Brucella melitensis* biovar 3, Spontaneous abortion, Human brucellosis

## Abstract

**Background:**

Brucellosis is a worldwide zoonotic disease caused by *Brucella* spp. *Brucella* invades the body through the skin mucosa, digestive tract, and respiratory tract. However, only a few studies on human spontaneous abortion attributable to *Brucella* have been reported. In this work, the patient living in Shanxi Province in China who had suffered a spontaneous abortion was underwent pathogen detection and *Brucella melitensis* biovar 3 was identified.

**Case presentation:**

The patient in this study was 22 years old. On July 16, 2015, she was admitted to Shanxi Grand Hospital, Shanxi Province, China because of one day of vaginal bleeding and three days of abdominal distension accompanied by fever after five months of amenorrhea. A serum tube agglutination test for brucellosis and blood culture were positive. At the time of discharge, she was prescribed oral doxycycline (100 mg/dose, twice a day) and rifampicin (600 mg/dose, once daily) for 6 weeks as recommended by the World Health Organization (WHO). No recurrence was observed during the six months of follow-up after the cessation of antibiotic treatment.

**Conclusions:**

This is the first reported case of miscarriage resulting from *Brucella melitensis* biovar 3 isolated from a pregnant woman who was infected through unpasteurized milk in China. Brucellosis infection was overlooked in the Maternity Hospital because of physician unawareness. Early recognition and prompt treatment of brucellosis infection are crucial for a successful outcome in pregnancy.

**Electronic supplementary material:**

The online version of this article (10.1186/s40249-018-0411-x) contains supplementary material, which is available to authorized users.

## Multilingual abstract

Please see Additional file 1 for translation of the abstract into the five official working languages of the United Nations.

## Background

Brucellosis is a worldwide zoonotic disease caused by *Brucella* spp. The *Law of the People’s Republic of China on Prevention and Treatment of Infectious Diseases* classifies it as a Class B infection. *Brucella* invades the body through the skin mucosa, digestive tract, and respiratory tract. Livestock infected with *Brucella* often undergo spontaneous abortion and infertility, and have low reproductive and survival rates. Humans infected with *Brucella* mainly manifest fever, sweating, fatigue, and arthralgia, and can also suffer damages to the nervous, circulatory, and reproductive systems [1]. However, only few studies on spontaneous abortion attributable to *Brucella* have been reported. In this work, a patient living in Shanxi Province in China who had suffered a spontaneous abortion underwent pathogen detection to analyse the genetic characteristics of the spontaneous abortion-related *Brucella* strain. This helps to provide a scientific basis for the prevention and control of *Brucella* infection in pregnant women.

## Case presentation

**Fig. 1:**
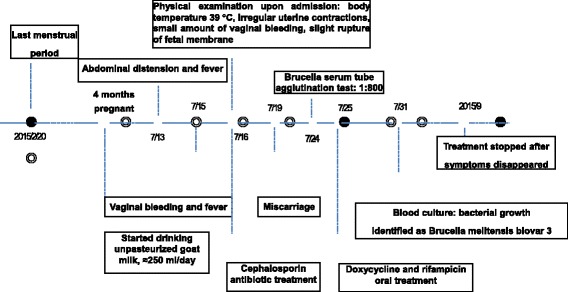
The onset and outcome of disease, diagnosis, and treatment

The patient in this study was 22 years old. She was admitted to Shanxi Grand Hospital, Shanxi Province, China, on July 16, 2015 because of one day of vaginal bleeding and three days of abdominal distension accompanied by fever after five months of amenorrhea. This patient had a history of regular menstruation, and her last menstrual period had been on February 20, 2015. An immunoassay showed her urine to be positive for human chronic gonadotrophin. The patient had no fever during early pregnancy and did not have a history of exposure to toxic, harmful, or radioactive materials. Down’s syndrome screening performed as part of a regular second-semester prenatal checkup showed no obvious fetal abnormality. The patient had abdominal distension with fever and received anti-infective treatment at a local hospital three days before coming to Shanxi Grand Hospital. One day before coming to Shanxi Grand Hospital, she suffered vaginal bleeding. She was given conventional tocolytic treatment, but the outcome was poor. The patient was examined after hospital admission and had a body temperature of 39 °C, pulse rate of 120 beats/min, breath rate of 21 breaths/min, and blood pressure of 90/53 mmHg, but no cardiopulmonary or abdominal abnormalities. Specialist examinations showed minor abdominal swelling, irregular contraction of the uterus palpable at two fingers under the uterus and umbilicus, and a small amount of vaginal bleeding. The fetal membrane was slightly ruptured, and the fetal heart rate was 170–180 beats/min. A complete blood count showed 16.6 × 10^9^/L white blood cells, 78.4% neutrophils, 16.5% lymphocytes, 4.9% monocytes, 3.63 × 10^12^/L erythrocytes, 106 g/L hemoglobin, 202.1 × 10^9^ g/L platelets, and 102.16 mg/L C reaction protein. Intravenous ceftriaxone (2 g/d), 25% magnesium sulfate, and antipyretic treatments were administered to the patient after her admission to Shanxi Grand Hospital, but the patient had a miscarriage and vaginal delivery of a female fetus on July 19. Her body temperature continued to fluctuate after admission, increasing to 39.3 °C the afternoon of July 19. Further questions about the patient’s medical history showed that this patient had sheep at home but never came into direct contact with them. However, she had begun to drink unpasteurized goat milk during her fourth month of pregnancy and was thus suspected of having *Brucella* infection. A serum tube agglutination test (SAT) for brucellosis and blood culture were immediately performed. The SAT result was 1:800, confirming brucellosis. This patient was given antibiotic treatment for three consecutive days. She was discharged from the hospital on July 24 because the fever stopped. At the time of discharge, she was prescribed oral doxycycline (100 mg/dose, twice a day) and rifampicin (600 mg/dose, once daily) for 6 weeks as recommended by the World Health Organization (WHO). No recurrence was observed during the six months of follow-up after the cessation of antibiotic treatment. The onset, diagnosis, and treatment of the disease in this patient are shown in Fig. 1.

### Serological testing

The diagnosis of brucellosis was based on the serum standard tube agglutination test (SAT). The SAT result was 1:800.

### Pathogen detection

Five milliliters of venous blood from the patient were collected and injected into a two-phase culture flask for culture. After detecting bacterial growth in the culture, traditional biological methods were used for the isolation and identification of the bacteria [2]. With the reference to the standard strain *B. melitensis* 16 M, colony morphology, Gram stain reaction, CO_2_ requirements, H_2_S production, inhibition of growth by basic Fuchsin and Thionin, agglutination with monospecific antisera, and phage lysis testing were performed. Serum and bacteriophage were provided by the Brucellosis Laboratory, National Institute for Communicable Disease Control and Prevention, and the Chinese Center for Disease Control and Prevention.

Specific sequences of the 16 MLVA primers are described in previous work [3]. The reaction system for genotyping included 10 μl 2 × *Taq* PCR Mastermix, 0.4 μl each of the 10 pmol/μl primers, and 1 μl DNA template, with sterile distilled water to a total volume of 20 μl. The amplification conditions were: 95 °C denaturation; 40 cycles of denaturation at 95 °C for 30 s, annealing at 60 °C for 30 s, and elongation at 72 °C for 30 s. Amplification products were analysed by microsatellite sequencing to convert the repeated unit according to the size of the PCR products. BioNumerics (Version 5.0) software was used for cluster analysis to perform an online comparison between the typing and the *Brucella* database. Nucleic acid extraction was performed using a bacterial whole genome nucleic acid extraction kit [Tiangen Biotech (Beijing) Co., Ltd., Beijing, China]. MLVA primers were synthesized by Sangon Biotech (Shanghai) Co., Ltd. (Shanghai, China), and STR microsatellite sequencing was performed by Tianyi HuiYuan Biotech Co., Ltd. (Beijing, China).

Seven housekeeper genes (*dnaK*, *gyrB*, *trpE*, *aroA*, *cobQ*, *gap*, and *glk*), one outer membrane protein gene (*omp25*), and one intergenic region *int-hyp* were used as the target genes of MLST for synthesis of the corresponding primers and for PCR [4]. PCR products were purified and subjected to bidirectional sequencing. The sequencing was completed by Tianyi HuiYuan Biotech Co., Ltd.. The tested sequences were compared to the sequences of allelic genotypes of the corresponding genes. The MLST online tool (http://pubmlst.org/perl/mlstanalyse/mlstan-alyse.=pubmlst) was used to analyse the alleles in the sequence.

## Results

Five milliliters of whole blood were extracted from the patient on July 20 and were found to have bacterial growth on July 26. The colonies were collarless and transparent, round in shape, and with smooth surfaces. Conventional identification by microscopy showed colonies to be gram-negative short bacilli that did not produce hydrogen sulfide and had positive monospecific antisera agglutination. The basic Fuchsin and Thionin tests and the bacteriophage Bk test were positive, while the Tb and Wb tests were negative, indicating that the colony was *B. melitensis* biovar 3, commonly found in sheep and goats. For MLVA-16 typing (Additional file 2: Table S1), panel 1 showed the sample to be a type 42 (1–5–3-13-2-2-3-2), belonging to the Eastern Mediterranean type; panel 2 typing showed the sample to be a 4–40–8-4-4-3-8-5, which was completely identical to the goat type 3 *Brucella* (2012167) strain in MLVA genotyping [5]. For MLST, the ST allele spectrum was 3–2–3-2-1-5-3-8-2, and MLST sequence typing was ST8 (Additional file 3) , which is a common sequence type found in China [6].

## Discussion and conclusions

Spontaneous abortion is a common complication of brucellosis in animals. The infection tends to localize to the placenta, which is associated with erythritol (a bovine growth stimulant). Although erythritol is not present in human placental tissues, brucellosis can lead to spontaneous abortion in human, especially in early pregnancy [7]. Khan et al. studied 92 cases of brucellosis during pregnancy in a hospital in Saudi Arabia during 1983–1995 and found a rate of spontaneous abortion in the first and second trimesters of 43% [8]. Roushan et al. studied 19 cases of brucellosis during pregnancy in the Babol region in Iran and observed 10 cases of spontaneous abortion, accounting for 53% of all cases [9]. Al-Tawfiq et al. reviewed the literature covering brucellosis during pregnancy from 1954 to 2011 and found that the incidence of spontaneous abortion and stillbirth among 430 cases ranged from 31 to 46%, which was much higher than in other pregnant women [10]. However, Gulsun et al. conducted a case-control study on brucellosis during pregnancy from 2003 to 2010 and showed no significant differences in fetal congenital malformations and/or mortality between patients infected with *Brucella* and the control group, but *Brucella* did cause premature birth and low birth weight [11]. The present case study of brucellosis-induced spontaneous abortion in the second trimester provides clinical evidence for miscarriage caused by *Brucella* infection. The *B. melitensis* biovar 3 isolated from the blood culture belonged to the dominant strain found in Shanxi Province. Further study of the mechanism underlying miscarriage caused by *Brucella* will be necessary, and genome sequencing is in progress.

Milk from cattle, goats, and other animals with brucellosis contains large numbers of *Brucella*. It is possible to acquire brucellosis through the consumption of unpasteurized milk and dairy products [12]. The symptoms of brucellosis are atypical, and cases are easily misdiagnosed. In this study, the patient was treated in our hospital due to miscarriage and atypical symptoms of brucellosis. However, during her hospitalization, the patient did not immediately mention consuming goat’s milk. Although our staff had been actively looking for the cause of the fever, we only suspected *Brucella* infection after the patient’s miscarriage. We confirmed the diagnosis five days after her admission to the hospital.

It is difficult for antibiotics and antibodies to enter cells, so single-drug therapy cannot completely eliminate the bacteria. The WHO Expert Committee recommends brucellosis be treated using a combination of doxycycline (200 mg oral admission daily) and rifampicin (600–900 mg oral admission daily) for six weeks [7]. In this study, the patient was given combination therapy of doxycycline and rifampicin for six weeks and showed no recurrence during follow-up. The basic factor in the treatment of brucellosis is to ensure the effectiveness and adequate course of antibiotic treatment. Patients are urged to complete their full course.

In summary, this is the first reported case of miscarriage resulted from *Brucella melitensis* biovar 3 isolated from a pregnant woman who was infected through unpasteurized milk in China. Brucellosis infection was easily overlooked in the Maternity Hospital because of physician unawareness. The early recognition and prompt treatment of brucellosis infection are crucial for a successful outcome in pregnancy.

## Additional files

Additional file 1: Multilingual abstracts in the five official working languages of the United Nations. (PDF 502 kb)

Additional file 2: **Table S1.** Product size and repeat unit of 16 loci. (DOCX 68 kb)

Additional file 3: ST sequence data of 9 genes. (DOCX 17 kb)

## Abbreviations

MLST: Multilocus sequencing typing
MLVA: Multiple-locus variable number tandem repeat analysis
ST: Sequencing typing
