# Supplementary material for: A case report of spontaneous abortion caused by Brucella melitensis biovar 3
Source: Infect Dis Poverty. 2018 May 2;7:31. doi: 10.1186/s40249-018-0411-x (PMC5930816; doi:10.1186/s40249-018-0411-x)
Supplement: Supplementary file 1 — Multilingual abstracts in the five official working languages of the United Nations. (PDF 502 kb) [file 40249_2018_411_MOESM1_ESM.pdf]

Translation of the abstract into the five official working languages of the United Nations

تقرير الحالة للإجهاض التلقائي الذي تسببه البروسيلا المالطية بيوفار 3

هونغ-تشيا يانغ، جون-جون فينغ، كيو-تشانغ زانغ، روي هاو، سو-شيا ياو، رونغ زاو، دونغ-ري، بياو، بو-يون وي وهاي جيانغ

الملخص:
خلفية علمية: داء البروسيلات هو مرض عالمي حيواني المصدر تسببه البروسيلا متعددة الفصائل. البروسيلا تغزو الجسم عبر الأغشية المخاطية للجلد، الجهاز الهضمي والجهاز التنفسي. مع ذلك، بعض الدراسات القليلة فقط حول الإجهاض التلقائي عند الإنسان التي يُمكن نسبها إلى البروسيلاقد تم تقريرها. في هذا العمل، خضعت مريضةٌ تقطن في مقاطعة شانجي في الصين للكشف عن العامل المسبب المرض، وقد عانت من إجهاض تلقائي. البروسيلا المالطية بيوفار 3 هي التي تم تحديدها في تلك العملية.
عرض الحالة: تبلغ المريضة في هذه الدراسة اثني وعشرين عاماً. تم قبولها في مشفى شانجي الكبير، مقاطعة شانجي، الصين في السادس عشر من تموز عام 2015 وذلك بسبب إصابتها بيومٍ كاملٍ من النزيف في المهبل وثلاثة أيامٍ من الانتفاخ البطني تُرافقها الحمى بعد خمسة أشهر من انقطاع الحيض لديها. كانت اختبارات تراص أنبوب المصل للبروسيلات واستنبات الدم إيجابية. حين تم إخراجها من المشفى، وُصفت لها مضادات حيوية وهي دوكسي سيكلين (100 مغ/جرعة، مرتان يومياً) وريفامبيسين (600 مغ/جرعة، مرة واحدة يومياً) وذلك لستة أسابيع كما تنصح بها منظمة الصحة العالمية (WHO). لم تُلاحظ عودةٍ للمرض خلال ستة أشهرٍ تبعت قطع العلاج بالمضادات الحيوية.
الخلاصة: هذا هو تقرير الحالة الأول للإجهاض الناتج عن البروسيلا المالطيةبيوفار 3 التي تم عزلها من امرأةٍ حُبلى أُصيبت بها عن طريق الحليب غير المبستر في الصين. خضعت الإصابة بالبروسيلات للإشراف من قبل مشفى التوليد بسبب عدم وعي الطبيب. إن الكشف المبكر والعلاج الفوري للإصابة بالبروسيلات مهمٌّ جداً وحاسم من أجل ناتجٍ ناجحٍ للحمل.

Translated from English version into Arabic by Yousra Fakhrey, through

TRANSLATORS
WITHOUT BORDERS

一例羊种 3 型布鲁氏菌致人自然流产的病例报道

杨红霞，冯军军，张秋香，郝瑞娥，姚素霞，赵嵘，朴东日，崔步云，姜海

摘要
引言：布鲁氏菌病，是由布鲁氏菌属的细菌侵入机体引起的一种人兽共患传染性疾病。布鲁氏菌可以通过皮肤粘膜、消化道、呼吸道侵入机体。布鲁氏菌感染引起人流产的相关研究却很少，本研究对山西省的一例布鲁氏菌致人自然流产病例进行病原学检测，经鉴定该病原为羊种 3 型。
临床资料：患者，女，22 岁，于 2015 年 7 月 16 日以“停经 5 月，下腹憋胀伴发热 3 天，阴道出血 1 天”收住山西大医院。追问病史，患者家中有羊，她本人未直接接触羊，但在孕 4 个月时开始喝未彻底灭菌的羊奶，故怀疑布鲁氏菌感染。经试管凝集试验（SAT）及血培养，确诊为布鲁氏菌病。出院后按照世界卫生组织推荐的治疗方案，给予口服多西环素（100 mg/次，每天 2 次）及利福平（600 mg/次，每天 1 次）治疗 6 周后停药。停药后随访半年未复发。
结论：这是首次经病原学证实的由于饮用未经巴氏消毒的羊奶感染羊种 3 型布鲁氏菌导致的自然流产病例报道。在布病流行区，妇产医院大夫应该对流产孕妇增加布病的检测，早期

诊断和治疗是避免孕妇流产的关键措施。

Translated from English version into Arabic by Hong-Xia Yang

Rapport sur un cas d'avortement spontané provoqué par*le biovar 3* de Brucella melitensis

Hong-Xia Yang, Jun-Jun Feng, Qiu-Xiang Zhang, Rui-E Hao, Su-Xia Yao, Rong Zhao, Dong-Ri, Piao, Bu-Yun Cui and Hai Jiang

Résumé:
Rappel des faits : La brucellose est une maladie zoonotique provoqué par des bactéries du genre *Brucella* spp. *Le brucella* se propage à travers la peau ou la muqueuse, les voies digestives et les voies respiratoires. Toutefois, Il n'existe que de rares cas d'avortement spontané qui, imputables au *Brucella* chez l'humain, ont été signalés. Dans cette étude, une patiente issue de la province de Shanxi en Chine, qui avait subi un avortement spontané, a été soumise à une détection de pathogènes. *Le biovar 3 de Brucella melitensis* a été identifé.
Présentation du cas: Dans ce rapport, la patiente était âgée de 22 ans. Elle a été admise à l'hôpital de Shanxi, dans la province de Shanki, en Chine, le 16 juillet 2015 en raison de saignements vaginaux pendant 24 heures et d'une distension abdominale durant trois jours accompagnée de fièvre après cinq mois d'aménorrhée. L'épreuve de séro-agglutination en tube pour la détection de la brucellose ainsi qu'une hémoculture se sont révélées positifs. A sa sortie de l'hôpital, son médecin lui avait prescrit par voie orale la doxycycline (100 mg/dose , deux fois par jour) et la rifampicine (600 mg/dose, une fois par jour) pour six semaines, comme recommandé par l'Organisation Mondiale de la Santé (OMS). Aucune réapparition des symptômes n'a été observée au cours de six mois de suivi à compter de l'arrêt du traitement antibiotique.
Conclusions : Il s'agit du premier cas signalé d'une fausse-couche imputable à un biotype isolé qui s’est avéré être du *biovar 3 de Brucella melitensis* chez une femme enceinte. Cette patiente a été contaminée par du lait non pasteurisé en Chine. La brucellose a été négligée à la Maternité de l'hôpital en raison de l'ignorance des praticiens. La détection rapide et le traitement efficace de la brucellose sont primordiaux pour la réussite de la grossesse.

Translated from English version into French by Vero Marie and Minh Bui, through

TRANSLATORS
WITHOUT BORDERS

Клинический случай самопроизвольного аборта, вызванного *Brucella melitensis,* биотип 3

Хун-Ся Ян, Цзюнь-Цзюнь Фэн, Цю-Сян Чжан, Жуй-Э Хао, Су-Ся Яо, Жун Чжао, Дун-Жи, Пяо, Бу-Юнь Цуй и Хай Цзян

Аннотация:

Краткое описание. Бруцеллёз представляет собой распространённое во всём мире зоонозное заболевание, вызываемое различными видами *бруцелл*. *Бруцеллы* проникают в организм через слизистую оболочку кожи, желудочно-кишечный тракт, а также дыхательные пути. Тем не менее, по данным исследований лишь некоторые случаи самопроизвольного аборта у людей обусловлены *бруцеллами*. В настоящей работе возбудитель был обнаружен у пациентки из провинции Шаньси в Китае, у которой случился самопроизвольный аборт. Был выявлен *биотип 3* Brucella melitensis.

Представление клинического случая. Возраст пациентки, включённой в данное исследование, составлял 22 года. 16 июля 2015 года она была доставлена в больницу Shanxi Grand Hospital в провинции Шаньси, Китай из-за однодневного влагалищного кровотечения и трёхдневного растяжения брюшной полости, которые сопровождались повышенной температурой после пяти месяцев аменорреи. Серологическая реакция агглютинации в пробирках на бруцеллез и гемокультура дали позитивные результаты. При выписке пациентке был назначен 6-ти недельный курс перорального доксициклина (100 мг/дозу два раза в день) и рифампицина (600 мг/дозу один раз в день) в соответствии с рекомендациями Всемирной организации здравоохранения (ВОЗ). За период шестимесячного обследования после завершения лечения антибиотиками рецедива не наблюдалось.

Выводы. Это первый зарегистрированный случай самопроизвольного аборта, обусловленного биотипом 3 *Brucella melitensis,* изолированным у беременной женщины, заражение которой произошло в результате употребления непастеризованного молока в Китае. Заражение бруцеллёзом не было выявлено в родильном доме вследствие медицинской неосведомлённости персонала. Распознавание на ранней стадии и оперативное лечение инфекции бруцеллёза играют решающую роль для успешного протекания беременности.

Translated from English version into Russian by Natalia Potashnik and Liudmila Tomanek, through

TRANSLATORS
WITHOUT BORDERS

Caso clínico de aborto espontáneo causado por *Brucella melitensis* biovar 3

Hong-Xia Yang, Jun-Jun Feng, Qiu-Xiang Zhang, Rui-E Hao, Su-Xia Yao, Rong Zhao, Dong-Ri, Piao, Bu-Yun Cui and Hai Jiang

Resumen:

Antecedentes: La brucelosis es una enfermedad zoonótica presente en el mundo entero y causada por la *Brucella* spp. La*Brucella* invade el cuerpo a través de la mucosa de la piel, el tracto digestivo y el tracto respiratorio. Sin embargo, se han informado solo unos pocos estudios de abortos espontáneos en humanos atribuibles a la *Brucella*. En este trabajo, se realizó un estudio de detección de patógenos a una paciente que vive en la provincia Shanxi en China y que había sufrido un aborto espontáneo. Se detectó la presencia de *Brucella melitensis* biovar 3.

Presentación del caso: La paciente de este estudio tenía 22 años. Fue ingresada al Grand Hospital de Shanxi, en la provincia de Shanxi, China, el 16 de julio de 2015 luego de un día con sangrado vaginal y tres días con distensión abdominal acompañada de fiebre, luego de cinco meses de amenorrea. La prueba de seroaglutinación en tubo para brucelosis y el cultivo de sangre dieron positivo. En el momento del alta se le recetó doxycyclina (100 mg/dosis, dos veces por día) y rifampicina (600 mg/dosis, una vez por día) durante 6 semanas, tal como recomienda la Organización Mundial de la Salud (OMS). No se observó la reaparición de la infección durante los seis meses posteriores al término del tratamiento con antibióticos.
Conclusiones: Este es el primer caso informado de aborto espontáneo como resultado de una infección por *Brucella melitensis* biovar 3 aislada de una mujer embarazada en China que se infectó al consumir leche sin pasteurizar. En el hospital maternal no identificaron la infección de brucelosis debido al desconocimiento de los médicos. La detección precoz y el tratamiento rápido de la infección de brucelosis son cruciales para que el embarazo resulte exitoso.

Translated from English version into Spanish by Soledad Lescano and Camila Kohen, through

TRANSLATORS
WITHOUT BORDERS
