# Supplementary material for: A case report of spontaneous abortion caused by Brucella melitensis biovar 3
Source: Infect Dis Poverty. 2018 May 2;7:31. doi: 10.1186/s40249-018-0411-x (PMC5930816; doi:10.1186/s40249-018-0411-x)
Supplement: Supplementary file 2 — Table S1. Product size and repeat unit of 16 loci. (DOCX 68 kb) [file 40249_2018_411_MOESM2_ESM.docx]

**Physical examination upon admission: body temperature 39 °C, irregular uterine contractions, small amount of vaginal bleeding, slight rupture of fetal membrane**

**Last menstrual period**

**Abdominal distension and fever**

**Brucella serum tube agglutination test: 1:800**

2015/9

**4 months pregnant**

7/31

7/15

7/25

7/19

● ◎ ◎ ◎ ◎ ● ◎ ◎ ● ◎

**Treatment stopped after symptoms disappeared**

7/13

7/24

7/16

2015/2/20

**Blood culture: bacterial growth**

**identified as Brucella melitensis biovar 3**

**Vaginal bleeding and fever**

**Miscarriage**

**Doxycycline and rifampicin oral treatment**

**Started drinking unpasteurized goat milk, ≈250 ml/day**

**Cephalosporin antibiotic treatment**
