# Supplementary material for: A case report of spontaneous abortion caused by Brucella melitensis biovar 3
Source: Infect Dis Poverty. 2018 May 2;7:31. doi: 10.1186/s40249-018-0411-x (PMC5930816; doi:10.1186/s40249-018-0411-x)
Supplement: Supplementary file 3 — ST sequence data of 9 genes. (DOCX 17 kb) [file 40249_2018_411_MOESM3_ESM.docx]

>dnak_G1

GGCATCGACCTGAAGAACGACAAGCTTGCCCTGCAGCGCCTCAAGGAAGC

TGCCGAAAAGGCCAAGATCGAACTGTCGTCCTCGCAGCAGACCGAAATCA

ACCTGCCGTTCATCACGGCTGACCAGACTGGCCCGAAGCATCTGGCGATC

AAGCTGTCGCGCGCCAAGTTTGAAAGCCTGGTCGATGATCTCGTGCAGCG

CACGGTCGAGCCGTGCAAGGCGGCGCTCAAGGATGCCGGCCTCAAGGCTG

GCGAAATTGACGAAGTGGTTCTGGTCGGCGGCATGACCCGCATGCCCAAG

ATTCAGGAAGTCGTGAAGGCCTTCTTCGGCAAGGAACCGCACAAGGGCGT

GAACCCGGATGAAGTCGTGGCCATGGGCGCGGCGATCCAGGGCGGCGTTT

TGCAGGGCGACGTCAAGGACGTGCTGCTGCTCGACGTGACCCCGCTTTCG

CTCGG

>gyrB_G1

CGATCAGGTGGGCACCCTCATCACGGCGCTTGGCACCTCCATCGGCAAGG

ATGAAACGCACGGCTTCAACGCCGACAAGCTGCGTTATCACAAGATCATC

ATCATGACCGACGCCGACGTCGATGGCGCCCATATTCGTACGCTTCTGCT

CACCTTCTTCTTCCGGCAGATGCCGGAACTGATCGAACGCGGGCATATCT

ATATCGCGCAGCCGCCGCTCTATAAGGTGACACGCGGCAAGTCTTCGCAA

TATATCAAGAACGAAGCCGCCTTTGAGGATTTCCTCATCGAAACCGGCCT

TGAAGAAACGACACTGGAACTGGTGACTGGCGAAATGCGCGCCGGGCCGG

ATTTGCGCTCGGTGGTGGAGGATGCGCGCACGCTGCGTCAGCTTCTGCAC

GGCCTGCACACCCGCTATGACCGCAGCGTGGTGGAACAGGCGGCAATTGC

CGGCCTGCTCAACCCCGATGCCTCAAGGGACAATG

>trpE_G1

GCGCATTGGTATGGCGGCGCGATCGGCATGATGCATTTCAATGGCGATAT

GAATACAGGGCTGACGCTGCGCACCATCCGCATCAAGGATGGTGTGGCGG

AAATCCGTGCAGGGGCGACGCTTCTGTTCGATTCCAACCCTGACGAGGAA

GAAGCCGAGACCGAATTGAAGGCATCGGCCATGATTGCGGCTGTGCGGGA

CGCACAGAAGAGCAATCAGATCGCGGAAGAAAGTGTGGCGGCAAAGGTGG

GTGAGGGGGTTTCGATCCTGCTGGTCGATCACGAGGATTCCTTCGTCCAT

ACGCTTGCCAATTATTTCCGCCAGACGGGCGCCAAGGTTTCCACCGTGCG

TTCACCGGTGGCAGAGGAGATATTCGACCGCGTCAATCCCGATCTGGTGG

TGTTATCGCCGGGACCGGGCTCGCCGCAGGATTTCGATTGCAAGGCGACC

ATCGATAAGGCGCGCAAGCGCCAGCTTCCGATTTTTGGCGTCTGCCTCGG

CCTTCAGGCCCTGGCGGAAGCCTATGGGCGGC

>aroA_G1

TGCCGGGTGATCCCTCGTCAACGGCTTTTCCGCTGGTGGCCGCCCTTCTG

GTCGAAGGTTCGGACGTCACCATCCGCAATGTGCTGATGAACCCGACCCG

CACCGGCCTGATCCTGACGTTGCAGGAAATGGGGGCGGATATCGAGATCA

TCGATCCACGCCTTGCCGGCGGCGAGGATGTCGCCGATCTGCGCGTCAAG

GCCTCGAAGCTGAAAGGCGTTGTCGTTCCGCCGGAACGTGCGCCTTCGAT

GATCGATGAATATCCGGTTCTGGCCATTGCCGCGTCTTTTGCGGAAGGCG

AAACCGTGATGGACGGTCTCGATGAACTGCGCGTCAAGGAATCGGATCGT

CTGGCGGCCGTTGCGCGCGGCCTTGAAGCCAATGGTGTCGATTGTACCGA

AGGCGAGATGTCGCTGACGGTTCGTGGCCGCCCCGGCGGCAAGGGGCTGG

GCGGTGGCACGGTTGCAACCCACCTCGACCACCGCATCGCGATGAGTTTC

CTCGTCATGGGCCTTGCATCGGAAAAGCCGGTTACGGTGGATGACAGCAC

CATGATCGCCACCTCTTTCCCGGAATTCATGGGCCCTGATG

>cobQ_G1

CGGGTTTCAAATGCTTGGACGGCGGATCAGTGACCCGGCGGGTATTGAAG

GCAATGTGCGCGATATCGAGGGGCTGGGCCTTCTCGATATCGAGACGATG

ACGGAGCCGGAAAAAGTGGTTCGCAATGTTGAGGCGGTGTCGCTGCTGCA

TGATGAGCCGCTGGAGGGCTATGAAATCCACATCGGGCGCACCAGCGGGC

CGGATATGGCGCGGCCATTTGCGCGTATCGGCGATCATGATGATGGGGCC

GTCTCGCCCGATGGTCGTATCATGGGAACCTATCTCCACGGTGTTTTCAG

TGCGGATCGTTTCCGCCACCACTTTTTGCGCGCGCTGGGTGTGGAAGGCG

GTCAGATGAATTATCGCGAGAGCGTCGAAGAGGCTCTGGGCGAACTGGCT

GAAGGGCTGGAAGCCTCGCTGGATATTGATGGCCTGTTTGCGCTGGCATG

>gap_G1

CGTCATCGTCTCCGCTCCCGCAGACGGTGCCGATCTCACCGTCGTCTATG

GTGTCAACAACGACAAGCTGACGAAGGACCATCTGGTCATCTCCAACGCT

TCGTGTACCACCAACTGCCTTGCGCCGGTGGCTCAGGTTCTCAACGATAC

TATCGGTATCGAAAAGGGCTTTATGACCACGATCCACTCCTATACGGGCG

ACCAGCCGACGCTGGACACCATGCACAAGGATCTCTACCGCGCCCGCGCC

GCTGCCCTTTCCATGATCCCGACCTCGACGGGTGCGGCCAAGGCCGTCGG

TCTCGTTCTGCCGGAACTGAAAGGCAAGCTCTACGGCGTTGCCATTCGCG

TCCCGACCCCAAATGTCTCGGTCGTTGATCTCACCTTCATCGCCAAGCGT

GAAACCACCGTTGAAGAAGTCAACAATGCGATCCGCGAAGCCGCCAATGG

CCGCCTCAAGGGCATTCTCGGCTATACCGATGAGAAGCTCGTCTCGCACG

ACTTCAACCACGATTCCCATTCCTCGGTCTTCCACACCGACCAGACCAAG

GTTATGGACGGCACCATGGTGCGTATCCTGTCGTGGTACGACAATGAATG

GGGC

>glk_G1

AAGATCGGCGGCAAACCGGAGGAGGCTGTTGCCACCCGCGTCGTGCTCGG

CCCCGGCACGGGCCTTGGCGTGGCAGGTCTGGTTTGCACACGTCATGCAT

GGGTTCCGGTTCCCGGTGAAGGCGGTCATATCGATATCGGTCCACGCACC

GAACGCGACTACCAGATTTTCCCGCATATCGAACGCATCGAAGGGCGTGT

CACCGGCGAGCAAATTCTTAGCGGGCGGGGCCTGCGCAACCTCTATCTGG

GCATCTGCGCGGCCGACAAGATCACGCCCACCCTTGAGACGCCAGTAGAC

ATTACATCCGCCGGACTGGACGGCAGCAATCCACAAGCCGCAGAAACGCT

TGACCTCTTCGCCACCTATCTGGGGCGGCTTGCGGGCGACCTTGCGCTCA

TTTTCATGGCGCATGGCGGCGTTTATCTTTCGGGTGGCATCCCGGTGCGC

ATCCTTTCCGCCCTCAAGGCCGGTTCGTTCCGCGCAGCCTTCGA

>omp25_G1

TCTTAAGTCTCTCGTAATCGTCTCGGCTGCGTTGCTGCCGTTCTCTGCGA

CCGCTTTTGCTGCCGACGCCATCCAGGAACAGCCTCCGGTTCCGGCTCCG

GTTGAAGTAGCTCCCCAGTATAGCTGGGCTGGTGGCTATACCGGTCTTTA

CCTTGGCTACGGCTGGAACAAGGCCAAGACCAGCACCGTTGGCAGCATCA

AGCCTGACGATTGGAAGGCTGGCGCCTTTGCTGGCTGGAACTTCCAGCAG

GACCAGATCGTATACGGCGTTGAAGGTGATGCAGGTTATTCCTGGGCCAA

GAAGTCCAAGGACGGCCTGGAAGTCAAGCAGGGCTTTGAAGGCTCGCTGC

GTGCCCGCGTTGGCTACGACCTGAACCCGGTTATGCCGTACCTCACGGCT

GGTATTGCCGGTTCGCAGATCAAGCTTAACAACGGCTTGGACGACGAAAG

CAAGTTCCGCGTGGGTTGGACGGCTGGTGCCGGTCTCGAAGCCAAGCTGA

CGGACA

>ink_hyp_G1

CAACTACTCTGTTGACCCGATAACGGTGGTATCATGCCGAGTAAAAGAGC

GATCTTTACACCCTTGTCGATCCTGTTTCGCCCCCGCCACAACACAGCCT

GATCGGCAAGCTGTGCTTTGGTGGAGGCGCCGGGTACCGCCCCCGGGTCC

AATGGGTTTATTACACCGTCCGTTTATCACCATAGTCGGCTTGCGCCGAC

AGGACGTATATAGGCGTGGTTTTTACCGATTGGAAGGGGGCTTGTGCGTT

TTCGCGCAAGACCGACAGAGGTGGTGCGGCCCTTCCGTTCATTTTCCATT

GACAGCTTCCGCGCGCTGGTCAATCCTCACAATATATCGGGATCGGCCTT

GAAGAGGCTTGGCGCAGCCGGGGCGGAAACCATGGCTGAAACGGGGACGA

TATGCCCCATTCGAAGGAGAGTGGATATATGAGTGAATATCTCGCGGATG

TCCGTCGC
